# Multi-Stage Corn-to-Syrup Process Monitoring and Yield Prediction Using Machine Learning and Statistical Methods

**DOI:** 10.3390/s24196401

**Published:** 2024-10-02

**Authors:** Sheng-Jen Hsieh, Jeff Hykin

**Affiliations:** 1 Department of Engineering Technology & Industrial Distribution and Department of Electrical & Computer Engineering, Texas A&M University, College Station, TX 77843, USA; 2 Department of Computer Science & Engineering, Texas A&M University, College Station, TX 77843, USA; jeff.hykin@tamu.edu

**Keywords:** machine learning, corn syrup, dextrose equivalent value, ANN, SVM, process control, prediction, noise tolerant

## Abstract

Corn syrup is a cost-effective sweetener ingredient for the food industry. In producing syrup from corn, process control to enhance and/or maintain a constant dextrose equivalent value (DE) is a constant challenge, especially in semi-automated/batch production settings, which are common in small to medium-size factories. Existing work has focused on continuous process control to keep parameter values within a setpoint. The machine learning method applied is for time series data. This study focuses on building process control models to enable semi-automation in small to medium-size factories in which the data are not as time dependent. Correlation coefficients were used to identify key process parameters that contribute to feed pH value and DE. Artificial neural network (ANN), support vector machine (SVM), and linear regression (LR) models were built to predict feed pH and DE. The results suggest (1) model accuracy ranges from 91% to 96%; (2) the ANN models yielded about 1% to 3% higher accuracy than the SVM and LR models and the prediction accuracy is robust even with as few as six data sets; (3) both the SVM and ANN models have noise tolerant properties, but ANN has a higher noise tolerance than SVM; (4) SVM performance can be hindered when using high-dimensional data sets; (5) the LR model yields higher variation in accuracy prediction than ANN and SVM; (6) distribution fitting is a good approach for generating data; however, fidelity of fitting can greatly impact accuracy; and (7) multi-stage models yield higher accuracy than single-stage models, but there are pros and cons to each approach.

## 1. Introduction

Corn syrup is an inexpensive sweetener that is widely used in processed foods. It is obtained by the hydrolysis of corn starch [1]. In the U.S. alone, on an annual basis, 10 billion pounds are used by consumers directly and another 15 billion are used in the preparation of processed foods such as soft drinks, cookies, and cakes [2]. Globally, the market for corn syrup was USD 9.8 billion in 2021; it is projected to reach USD 13.5 billion by 2031 [3].

Corn syrup production involves several stages, including milling, purification, starch conversion, evaporation, filtration, and sterilization. To facilitate automation, efficiency, and consistency in producing very large quantities of corn syrup, large factories typically use continuous production processes. However, batch production processes are often used when smaller quantities are needed, such as for the production of specific syrup types or for quality adjustments [4,5,6]. Batch production is often semi-automated and is used by small to medium manufacturers with limited infrastructure capital investment, a large product mix, smaller production quantities, and uncertainty in work-in-process product quality.

In both continuous and batch production settings, the ability to enhance and/or maintain a constant dextrose equivalent value (DE) throughout the process is essential to obtaining consistent high quality in the final product. For example, Omobuwajo [7] conducted a 24-factorial experiment to understand the effects of the particle size, hydration ratio, level of substitution of corn with malted sorghum, and level of exogenous alpha-amylase on conversion efficiency. Based on this work, Omobuwajo developed a model of the saccharification efficiency of the corn starch conversion process. It is not clear if the process studied was automated or how each parameter relates to specific processing steps and associated measurement sensors.

Machine learning techniques can be used to predict a target value. For example, Hsieh [8] used thermal profiles of electronic components as inputs to ANN and regression models designed to predict voltage stress. Their results suggest that the ANN model predicted stress levels more accurately than the regression models and was better able to tolerate the presence of noise in the data sets.

Machine learning models have also been used to predict DE. For example, Tong et al. [9] used LASSO analysis, genetic algorithms, and variable importance analysis in developing neural networks to model an automated process. Data from 20 distributed control system sites were used as inputs to a neural network model, with DE as the output. The prediction results from their models had a relative error of less than 0.1%. Similarly, Zhang et al. [10] used artificial neural networks, random forest, and extreme gradient boosting (XGBoost) machine learning methods to derive predictive variables in starch flow and sugar production. The neural network and XGBoost methods yielded higher accuracies than the random forest method.

Meng et al. [11] describe the design of a model predictive control system for the corn-to-sugar process using a machine learning model called Recurrent Neural Network Long Short-Term Memory. Their simulation studies suggested that their model could be used to successfully control DE at the target values under situations such as setpoint changes and disturbances.

Response surface methodology (RSM) is widely used in industry to understand how input parameters affect response factors via systematic experimentation. Gebru et al. [12] employed RSM to study sweet potato-based glucose syrup production and preservation. They found that hydrolysis time, sulfuric acid concentration, and temperature have a significant positive effect on glucose syrup yield. Recent work by Rezvanian [13] used RSM to study the sweet potato corn starch process. The intention was to investigate the effect of variables such as enzyme quantity and incubation time within a batch production process for sweet potato starch syrup. A quadratic model was developed for the saccharification step and was found to be statistically significant (<0.05).

Most existing work has focused on continuous process control to maintain parameter values around a setpoint. There has been less focus on batch production processes in which key process parameters—such as pH value—are checked at certain stages to determine if adjustments are needed to increase the overall yield rate at a target value. This type of process can be formulated as a multi-stage optimization problem.

The multi-stage optimization problem was first presented by Dantzig [14] in 1951 and has been applied in a variety of real-world applications, including supply chain planning [15], scheduling [16], and finance [17]. Dantzig incorporated an “uncertainty” factor into linear programming methods [14]. In a recent review, Bakker et al. [18] observed that multi-stage optimization problems are intended to address the fact that real-world applications often involve uncertain data and occur in a temporal context.

Moradi-Aliabadi and Huang [19] introduce a mathematical framework for optimal process sustainability performance enhancement. Economic, environmental, and social concerns, as well as technical feasibilities, are considered, where data uncertainty is dealt with by interval parameters. A genetic algorithm method and Monte Carlo simulation technique were employed for solution search. A comprehensive case study related to biodiesel manufacturing is used to illustrate the application of the framework.

Hsieh et al. [20] propose a multi-stage architecture for adaptive autonomous driving assistance. ANN, SVM, and RF models were built to provide advice to human drivers of autonomous vehicles. In Stage I, trust in automation is assessed based on vehicular data such as braking, use of cruise control, use of autopilot, and speed. In Stage II, appropriate advice is generated using speed, road condition, distraction, and trust in automation as inputs. Their results suggest that human trust in automation can be quantified and predicted with 80% accuracy based on vehicular data, and that adaptive speech-based advice can be provided to drivers with 90 to 95% accuracy using ANN and SVM models.

The objectives of this work are to (1) formulate the corn-to-syrup process as a multi-stage problem with quality check points, (2) identify parameters that contribute most to product yield, (3) develop prediction models that employ machine learning and statistical techniques, and (4) compare and understand different machine learning and statistical methods for yield prediction accuracy.

## 2. Materials and Methods

### 2.1. Data Source

An industry partner provided 370 data sets, which included values for 31 process parameters. Incomplete data sets were deleted, leaving 353 data sets. After conducting a preliminary screening of the data for redundancy, 17 parameters were used for this study. These parameters were further divided over two stages based on the process flow.

### 2.2. Formulate Multi-Stage Problem

Corn syrup is produced by combining corn starch with dilute hydrochloric acid, and then heating the mixture under pressure. The hydrochloric acid and heat break down the starch molecules and convert them into sugar [1].

To better formulate the corn syrup production process as a multi-stage problem, it is important to understand each processing step and quality check points. The quality check points can be used to divide the process into stages.

Typical production steps include (1) starch slurry preparation, (2) liquefaction/acid conversion, (3) enzyme liquefaction, (4) enzyme saccharification, (5) filtration and decolorization, (6) evaporation, and (7) final storage [4,5,6]. During steps 2 to 4, the starch slurry is converted into glucose syrup through a process called hydrolysis. Acids and enzymes are used to break down the starch molecules into simpler sugars. During these processing steps, the solution pH value is monitored and adjusted by adding more acid, enzymes, or starch. In the evaporation step, the temperature is monitored and controlled to concentrate syrup to reach the desired sweetness level [4].

Maintaining pH value is a key measurement from the early stages of production, starting from the corn-to-starch mix. Samples are taken in the middle of the process and examined in a lab to compare with the pH sensor readings. Additional acid, water, or starch may be added to the container to raise or lower the pH value of the solution. In the later stages of production, heat operations are introduced to energize the chemical DE process so that more sugar is generated from the process. With this understanding, we can divide the production process into two stages to facilitate the quality checking and modeling processes described in later sections of this document. In Stage I, the focus is on the pH value of the solution. In Stage II, the focus is on the dextrose equivalent.

**Figure 1:**
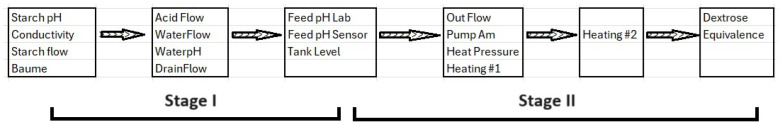
Distribution of parameters for Stages I and II.

Figure 1 shows 17 process parameters that are measured during a typical corn syrup production process. Stage I includes 11 process parameters: starch pH, conductivity, starch flow, baume, acid flow, water flow, water pH, drain flow, feed pH lab, feed pH sensor, and tank level. Stage II includes 9 process parameters: feed pH lab, feed pH sensor, tank level, out flow, pump amperage, heat pressure, heating unit #1, heating unit #2, and dextrose equivalent.

### 2.3. Yield Prediction Model Development

The modeling process was as follows:

- Identify performance measure variables for each production stage.
- Identify process parameters that strongly correlate with the performance measures.
- Apply machine learning and statistical techniques to identify process parameters that contribute strongly to the performance measures.
- Develop and evaluate models to predict performance measures such as feed pH lab and DE value.
- Fit the data into a distribution and generate data for modeling and testing.
- Further evaluate model accuracy using field data and other tests.

## 3. Results and Discussion

### 3.1. Identify Performance Measure for Each Production Stage

The pH values are monitored and controlled during the corn syrup production process, specifically in the liquefaction/acid conversion, enzyme liquefaction, and enzyme saccharification processing steps [4]. Therefore, pH (Feed pH Lab) is used as the performance measure for Stage I. Furthermore, because reaching a desired dextrose equivalent (DE) value is the goal of the production process, DE is used as the performance measure for Stage II.

### 3.2. Identify Process Parameters that Strongly Correlate with the Performance Measures

**Figure 2:**
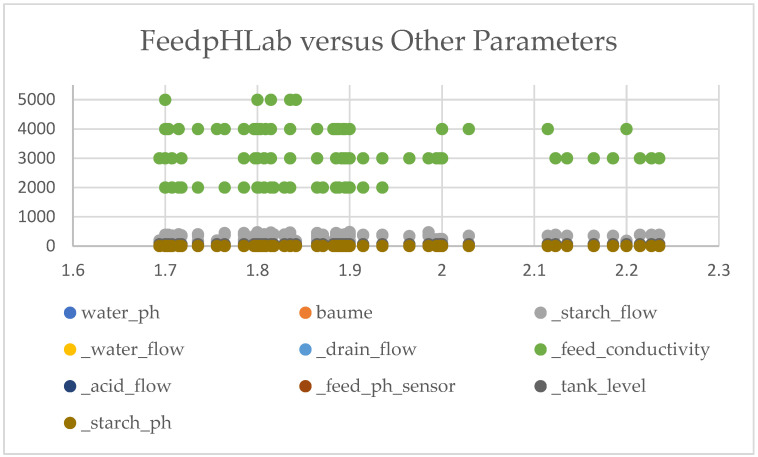
Scatter plot of FeedpHLab versus other parameters.

Scatter plots of the raw data of FeedpHLab and dextrose equivalent (DE) variables versus other parameters were first plotted to eyeball the relationship between the Stage I and II performance measures and other parameters. Figure 2 shows that (1) most of the parameters are separate from each other, (2) the relationship of FeedpHLab to other parameters is approximately linear, and (3) a few parameters are close to each other.

**Figure 3:**
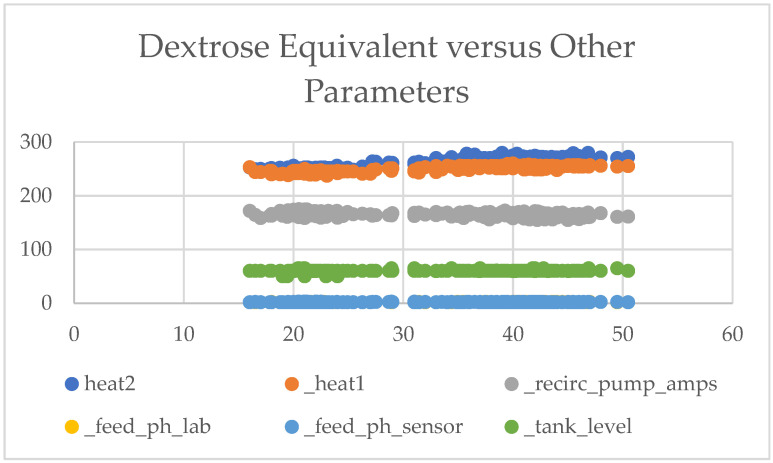
Scatter plot of dextrose equivalent (DE) versus other parameters.

Figure 3 shows (1) some lines are close to others, forming three distinct groupings, and (2) some parameters have a positive relationship to DE.

To further understand the relationship of FeedpHLab and dextrose equivalent with other parameters, correlation coefficients were calculated between the process parameters associated with Stages I and II (described in Figure 1) and the stage performance measure. Table 1 and Table 2 show the correlation coefficient of each process parameter associated with Stages I and II with their respective stage performance measure.

In Table 1, all the parameter correlation coefficients show weak relationships with the performance measure, FeedpHLab. The three parameters with the highest correlations with FeedpHLab are water pH, baume, and starch flow.

The correlation coefficient values of DE versus other Stage II parameters were also calculated. In Table 2, the parameters with the strongest correlation with DE are heat2, heat1, and recirc_pump_amps.

### 3.3. Apply Machine Learning and Statistical Techniques to Identify Process Parameters that Contribute Strongly to the Performance Measures

Machine learning modeling techniques—artificial neural networks (ANNs) and support vector models (SVMs)—were applied to model and predict FeedpHLab for Stage I and DE for Stage II. Model performance was then evaluated by randomly dividing the data into training and testing sets.

For the MATLAB ANN model, the Levenberg–Marquardt backpropagation algorithm (i.e., *trainlm function*) is applied to build a three-layer X-2-1-topology artificial neural network, where X refers to the number of input nodes corresponding to the number of parameters in each experiment described in Table 1 and Table 2. The 2 refers to two hidden layers and 1 refers to one output node. The Levenberg–Marquardt algorithm combines the steepest descent (far from the optimal values) and the Gauss–Newton methods (close to the optimal value), taking advantage of the high speed of the Gauss–Newton algorithm and the high stability of the steepest descent method [21].

For the MATLAB SVM regression model, a pretrained SVM regression model with the kernel function RBF was applied. The RBF kernel maps the input data into a higher-dimensional space where it becomes linearly separable. The SVM predicts the output based on the inputs corresponding to the experiment parameters described in Table 3 and Table 4.

For the statistical method, MATLAB’s *fitlm* linear regression model was employed to predict the performance measure (FeedpHLab for Stage I and DE for Stage II). A linear regression model describes the relationship between a dependent variable, Y, and one or more independent variables, X. In this case, Y is FeedpHLab and DE and X are the process parameters, such as water_pH and heat1.

Experiment Design and Results. The experiments were designed to reveal the effect of each parameter on performance measure. At the beginning of the experiments, the full set of parameters were used. Then, for each successive experiment, one parameter was eliminated from the experiment setup list. The parameters with stronger correlation coefficients will remain in the last experiment setup. Following this sequence, the experiments and results for Stages I and II are described as follows:

*Stage I*. Ten experiments were conducted. Each experiment involved building ANN, SVM, and LR models with a specific set of parameters. The parameters were chosen based on the correlation coefficient with the performance measure listed in Table 1. Table 3 shows, for each experiment, the prediction accuracy (Acc) for the Stage I performance measure (feedpHLab) and the parameters used in building the models for that experiment (indicated with an X). In this paper, prediction accuracy is defined as 1 − ((Pi − Ti)/Ti)%, where Pi is the predicted value for data sample I and Ti is the target value for data sample i. The accuracy values represent the average accuracy for a given number of samples (such as 353 data sets).

The results suggest that (1) the average accuracy of the ANN, SVM, and LR models are around 95%, 96%, and 96%, respectively; (2) the SVM model yields a lower variation of 0.19% throughout the experiments and the variations within ANN and LR are about the same at 1.3%; and (3) the experiment settings with the Starch Flow and Water pH parameters yield the highest accuracy for ANN and LR and approximate the highest accuracy for SVM (0.3 less than the highest). This suggests that the Starch Flow and Water pH parameters are good candidates for building the Stage I yield prediction model.

Statistical testing of the average accuracy of each method suggests (1) SVM_avg_ is significantly different than ANN_avg_, with a t_(α=0.025, n=9)_ value of −2.84; and (2) SVM_avg_ is not significantly different from LR_avg_, with a t_(α=0.025, n=10)_ value of −0.072. The ANN prediction model with Starch Flow and Water pH yields the highest accuracy among all the settings and comparisons with the SVM and LR models.

*Stage II*. Nine experiments were performed for Stage 2. Each experiment involved building ANN, SVM, and LR models with a specific set of parameters. Table 4 shows, for each experiment, the prediction accuracy for the Stage II performance measure (DH) and the parameters used in building the models for that experiment (indicated with an X). The results suggest (1) the average accuracies for the ANN, SVM, and LR models are around 94%, 93%, and 92%, respectively; (2) the ANN model yields the highest accuracy and exhibits lower variance than SVM and LR; and (3) the experiment setting with the Heat1, Heat2, Feed pH lab, and FeedpH Sensor parameters yields the highest accuracy in predicting the dextrose equivalent (DE) value. These four parameters are good candidates for Stage II prediction model formulation. Statistical testing of the average accuracy of each method suggests that (1) ANN_avg_ is significantly higher than SVM_avg_, with a t_(α=0.025, n=10)_ value of 3.50; and (2) ANN_avg_ is significantly higher than LR_avg_, with a t_(α=0.025, n=10)_ value of 4.10.

### 3.4. Develop and Evaluate Models to Predict Performance Measures

Continuing the machine learning modeling work described earlier, nonlinear machine learning modeling techniques (ANN and SVM) were applied to model and predict Feed pH lab for the Stage I process and DE for the Stage II process. LR models were not included in this step because their performance was not as good as that of the ANN and SVM models.

#### 3.4.1. Stage I—Machine Learning Model for Prediction of pH Value

For the Stage I (SI) ANN model, we applied a 2-2-1 ANN topology with Starch Flow and Water pH as the two input nodes, two hidden layers, and one output node, which was FeedpHLab. The same topology was applied for the SVM model. Model effectiveness was then evaluated by randomly dividing the data into two sets for training (2/3 of the data) and testing (1/3 of the data).

Table 5 shows the results from the SI_ANN_ and SI_SVM_ models. Prediction accuracy is defined as 1 − ((Pi − Ti)/Ti)%, where Pi is the predicted value for data sample I and Ti is the target value for data sample i. The accuracy values represent the average accuracy for a given number of samples (such as 353 data sets). The prediction accuracies for SI_ANN_ and SI_SVM_ were very close (µ = 96.39, SD = 0.70 versus µ = 96.49, SD = 0.77), but the SVM accuracies were slightly higher with greater variation. The accuracy did not increase much as sample size increased to 353 data sets.

#### 3.4.2. Stage II—Machine Learning Model for Prediction of DE

For the Stage II (SII) ANN model, we applied a 4-2-1 ANN topology with FeedpHSensor, FeedpHLab, Heat1, and Heat2 as the four input nodes, two hidden layers, and one output node, which was the DE value. The same topology was used for the SVM model. Model effectiveness was then evaluated by randomly dividing the data into testing and evaluation sets.

Table 6 shows the results from the SII_ANN_ and SII_SVM_ models. Prediction accuracy is defined as 1 − ((Pi − Ti)/Ti)%, where Pi is the predicted value for data sample I and Ti is the target value for data sample i. The accuracy values represent the average accuracy for a given number of samples (such as 353 data sets). The prediction accuracies for SI_ANN_ and SI_SVM_ were very close (µ = 95.80, SD = 0.76 versus µ = 96.52, SD = 0.73), but the accuracies of SVM were slightly higher with a smaller variation. The accuracy did not increase much as sample size increased to 353 data sets.

In short, we can conclude that appropriate parameters were selected for the Stage I and II machine learning models since the accuracy is above 95% even for a small number of data sets, such as six data sets. Also, the quality of the data is relatively uniform and consistent, which is why the accuracy did not increase or decrease significantly for different sample sizes (less than 2%—Max–Min).

Findings and Observations. From the above discussion and summary, we can conclude that (1) the correlation between the process parameters and the performance measure are useful indicators of fit for building prediction models; (2) Water pH and Starch Flow are the parameters for the Stage I model to predict Feed pH lab; (3) Heat1, Heat2, Feed pH lab, and Feed pH Sensor are the parameters for the Stage II model to predict the DE value; (4) ANN models for Stage I and II yield good (Stage I) to the best (Stage II) results as compared to SVM and LR models; and (5) the ANN model prediction accuracy is robust even with as few as six data sets. Observations include the following: (1) ANN model prediction performance variation decreases as the correlation between the performance measure and the parameters increases; for example, in Stage I, Water pH and Starch Flow have weak correlations with FeedpHLab. In Stage II, Heat 1 and Heat 2 have strong correlations with DE. The variance in the model’s prediction accuracy improves from 1.31% to 0.41%. (2) The variation in prediction accuracy for the LP models is the highest of the three model types regardless of the correlation of the parameter values with the performance measure.

### 3.5. Cross-Validation of Machine Learning Models Using K-Folds Method

Cross-validation is one of the most widely used data resampling methods for model selection and evaluation. It can be used to tune hyperparameters of statistical and machine learning models, to prevent overfitting, to compare learning algorithms, and to estimate the generalization error of predictive models [22]. In this paper, K-folds cross-validation was used to evaluate the accuracy of the machine learning methods described in the experiments in Table 3 and Table 4. K was set to 10 for both the ANN and SVM methods.

### 3.6. Fit the Data into a Distribution and Generate Data for Modeling and Testing

Data collected from the corn-to-syrup process were fitted into a suitable distribution and validated using a Chi-Squares test since the number of data sets is greater than 25. Specifically, the parameters identified for Stage I (Starch Flow and Water pH) and Stage II (Heat2, Heat1, FeedpHSensor and FeedpHLab) were fit into distributions, which were then used to generate data needed for testing and evaluation.

#### 3.6.1. Stage I Process Parameter Distribution

The following is a summary of the process parameter distribution for each parameter of interest.

- Starch Flow: The top distribution fitting candidate was Triangle distribution, and the distribution parameters are Triangle (49.74, 395.42, 487.49).
- Water pH: The top distribution fitting candidate was Normal (1.93, 0.32).

#### 3.6.2. Stage II Process Parameter Distribution

The following is a summary of the process parameter distribution for each parameter of interest.

- FeedpHLab: The top distribution fitting candidate was Exvalue (0.71, 0.19)
- FeedpHSensor: The top distribution fitting candidate was Exvalue (0.71, 0.19).
- Heat 1: The top distribution fitting candidate was Triangle (230.23, 255.26, 266.59).
- Heat 2: The top distribution fitting candidate was Triangle (248.09, 248.69, 284.02).

Note that although four parameters were used to construct the Stage II machine learning model, for the evaluation of noise tolerance, noise was added only to FeedpHLab in the Stage II data sets. Since FeedpHLab is the output of the Stage I model, this allowed us to investigate the cumulative impact of noise in Stage I on DE, which is the performance measure for Stage II.

### 3.7. Further Evaluate Model Accuracy Using Field Data and Other Tests

#### 3.7.1. Noise Tolerance and Sensitivity Analysis

To test the robustness of the developed models, we arbitrarily added noise to the data to see how the models would respond in terms of accuracy. Noise was added by multiplying the fitted distribution parameters (such as standard deviation) of the data sets by 5%, 10%, or 15%. Then, the distribution with new standardization was used to generate the data needed for the modeling and evaluation. For example, a 5% noise level was added using the Normal (µ,s) distribution: s _new_ 5% noise = (s _org_) * 1.05. If the fitted distribution had more than two parameters—such as a Triangle distribution, which has three parameters (min, most, max)—then all the parameters were adjusted to ensure the variance increased to 1.05-fold of the original variance value. In the Stage I model, noise was introduced to both parameters; in the Stage II model, noise was introduced only in the FeedpHLab parameter. Since FeedpHLab is the output of the Stage I model, we wanted to understand the cumulative impact of noise in Stage I on the Stage II performance measure (DE).

Table 7 shows the results for the two models for Stage I starting from no noise to 15% noise added. The results indicate the following:

(1) The overall accuracy of both models ranges from about 97.42 (Max) to 94.85 (Min) with the presence of up to 15% noise added to the two input parameters. This suggests that the parameters used for the input and output nodes of the machine learning models are both accurate and robust.

(2) Adding noise up to 15% decreased accuracy by about 0.01% and increased data variation by up to 0.1%, which is relatively small compared to the amount of noise added.

(3) Overall SVM model accuracy is about 0.1% higher than the ANN model and the variance is about the same, which is consistent with the results presented in Table 5.

(4) For both models, as the number of data sets increases, accuracy decreases and variance increases. For the ANN model, from no noise to a 15% noise level, the accuracy was reduced by 0.7% (96.73–96.03%) and the variance increased by 0.01% (0.10–0.09%). For the SVM model, the accuracy was reduced by 0.76% (96.79%–96.03%) and the variance increased by 0.04% (0.07%–0.03%). The magnitude of change is less than 1%, which suggests that the data sets are relatively uniform.

**Table 7:** Stage I models—noise tolerance property testing results.

Noise/Data Sets	No Noise	+5% Noise	+10% Noise	+15% Noise	No Noise (Max)	µ	SD
SIANN (15 data sets)	96.84	96.61	96.70	96.77	0.07	96.73	0.09
SISVM (15 data sets)	96.83	96.75	96.77	96.81	0.02	96.79	0.03
SIANN (23 data sets)	95.13	95.53	96.70	97.16	−2.03	96.13	0.83
SISVM (23 data sets)	97.42	97.14	97.22	97.45	−0.03	97.31	0.13
SIANN (53 data sets)	96.06	94.85	95.32	95.03	1.03	95.32	0.46
SISVM (53 data sets)	95.71	95.34	95.39	95.27	0.44	95.43	0.17
SIANN (153 data sets)	96.31	96.48	96.11	96.39	−0.08	96.32	0.14
SISVM (153 data sets)	96.27	96.19	96.22	96.30	−0.03	96.25	0.04
SIANN (253 data sets)	96.65	95.91	95.08	95.74	−0.09	95.85	0.56
SISVM (253 data sets)	95.59	95.80	95.60	95.82	−0.23	95.70	0.11
SIANN (353 data sets)	96.17	96.17	96.22	95.96	0.21	96.13	0.10
SISVM (353 data sets)	95.92	96.12	96.05	96.01		96.03	0.07
µ	96.24	96.07	96.12	96.23	Sub	0.60	0.01
SD	0.60	0.61	0.64	0.70	Sub	0.76	0.04
µANN	96.19	95.93	96.02	96.18			
SDANN	0.55	0.60	0.63	0.70			
µSVM	96.29	96.22	96.21	96.28			
SDSVM	0.65	0.59	0.63	0.70			

Table 8 shows the results for the two models for Stage II starting from no noise to 15% noise added. The results indicate the following:

(1) Column 1 (A–B) of Table 8 shows the difference in accuracy in the ANN and SVM models before and after replacing the FeedpHLab original data with the new data generated from the distribution fitting of the FeedpHlab parameter. The results suggest that the impact was as large as 39.19% and as small as 2.44%. The average impact was about 19.12% and the average variation was about 11.86%. This could mean that more data are needed to better fit the original data into the distribution. Alternatively, it could mean that FeedpHLab is a critical factor contributing to the performance measure DE. In any case, the use of FeedpHLab for modeling appears to be appropriate.

(2) When comparing model accuracy after adding between 5% and 15% noise for the same number of data sets, the variation is about 1.27%, which is small relative to the 15% noise added to the data set. This suggests that both the ANN and SVM models have noise-tolerant properties.

(3) The ANN model seems to have higher noise tolerance than the SVM model. When comparing the performance of the ANN and SVM models in terms of the values of µ_ANN_, SD_ANN_, µ_SVM_, and SD_SVM_, the mean of the ANN model is about 5.35% higher than that of the SVM (78.48–73.13%); however, the variation is about the same (SD_ANN_ = 11.91% and SD_SVM_ = 11.02%). The accuracy decreased by about 19.35% and the variation increased by about 9.28% due to the number of data sets used to model the Stage II process.

(4) For both models, as the number of data sets increases, accuracy increases and variance decreases. For the ANN model, the correlation between the number of data sets and the accuracy is 0.74, which is a strong correlation. In addition, the correlation between the number of data sets and the variation in accuracy is 0.60, which means that as the number of data sets increases, the variation in accuracy decreases. The SVM model also shows a correlation between the number of data sets and accuracy (0.47) and between the number of data sets and the variation in accuracy (−0.37).

**Table 8:** Stage II models—FeedpHLab parameter noise tolerance property testing results.

Noise/Data Sets	A-B	A: No Noise Added	B: New Dist	+5% Noise	+10% Noise	+15% Noise	No Noise (Max)	µ	SD
SII_ANN_ (10 data sets)	15.87	97.3	81.43	78.90	78.83	78.93	18.47	79.52	1.10
SII_SVM_ (10 data sets)	12.45	96.12	83.67	83.82	83.44	83.11	13.01	83.51	0.27
SII_ANN_ (15 data sets)	16.75	96.22	79.47	81.22	80.30	84.92	15.92	81.48	2.08
SII_SVM_ (15 data sets)	11.06	95.05	83.99	84.77	83.73	83.87	11.32	84.09	0.40
SII_ANN_ (23 data sets)	38.29	95.71	57.42	59.42	55.88	65.90	39.83	59.66	3.82
SII_SVM_ (23 data sets)	39.19	95.03	55.84	56.55	55.99	55.15	39.88	55.88	0.50
SII_ANN_ (53 data sets)	27.33	95.19	67.86	59.11	57.02	62.06	38.17	61.51	4.08
SII_SVM_ (53 data sets)	37.21	94.5	57.29	56.53	57.04	56.53	37.97	56.85	0.33
SII_ANN_ (153 data sets)	2.44	86.6	89.04	86.86	89.56	83.80	2.80	87.32	2.27
SII_SVM_(153 data sets)	21.73	97.3	75.57	74.27	75.08	74.27	23.03	74.80	0.56
SII_ANN_ (253 data sets)	6.49	96.12	89.63	91.01	90.15	89.55	6.57	90.09	0.58
SII_SVM_ (253 data sets)	18.31	96.22	77.91	77.49	77.78	77.49	18.73	77.67	0.18
SII_ANN_ (353 data sets)	4.01	95.05	91.04	89.87	89.04	89.19	6.01	89.79	0.79
SII_SVM_(353 data sets)	16.57	95.71	79.14	78.94	78.77	79.54	16.94	79.10	0.29
µ	19.12	95.15	76.38	75.63	75.19	76.02		75.80	
SD	11.86	2.50	11.73	12.07	12.60	11.17		11.78	1.09
µ_ANN_		94.60	79.41	78.06	77.25	79.19		78.48	
SD_ANN_		3.34	11.67	12.55	13.81	10.22		11.91	
µ_SVM_		95.70	73.34	73.20	73.12	72.85		73.13	
SD_SVM_		0.87	10.98	11.05	10.88	11.18		11.02	

#### 3.7.2. Sensitivity Analysis

**Figure 4:**
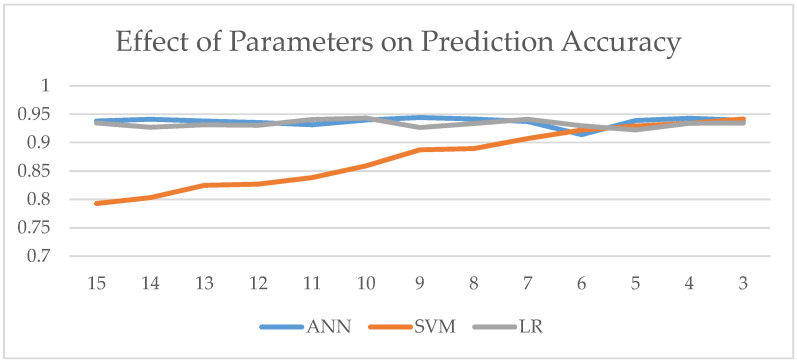
Effect of parameters on prediction accuracy.

A sensitivity analysis was performed to evaluate the performance of the machine learning methods as the number of parameters increases—in other words, how they would perform if *all* the process parameters were incorporated into the modeling process. In this experiment, 15 parameters involved in Stage I and II are first utilized as inputs to the models, with DE as the performance measure. Then, the number of parameters was reduced one at a time to see the effect of the number of parameters on the performance measure. In total, there were 13 experiments. K-folds (K = 10) were applied throughout the experiments. Figure 4 and Table 9 show the trend graph and accuracy values for each model and experiment.

The results suggest that (1) there is a strong negative correlation between the SVM model prediction accuracy and the number of parameters, meaning that SVM prediction accuracy decreases as the number of parameters increase; (2) for the ANN and LR models, the effect of the number of parameters on prediction accuracy is negligible; (3) ANN has the highest average accuracy and a lower variation than LR and SVM; however, ANN is only 0.4% more accurate than the LR model.

#### 3.7.3. Analysis of Single- Versus Multi-Stage Architecture for Yield Prediction

As described in the Introduction, a single-stage model for yield improvement is suitable for mature production and mass production environments in which setpoints for each process parameter are known. Data can be collected after a batch has been produced to provide feedback for minor process adjustments to maintain quality. However, in scenarios such as small batch production or the pilot production of a new product, the setpoints for process parameters are not fully understood. Therefore, multi-stage check points and process parameter adjustments are necessary to continue improving production yield.

Table 10 shows the accuracy of a multi-stage versus one stage. The results suggest that ANN and SVM models in the multi-stage yield a higher accuracy than in the single stage. The differences in accuracy range from 0.8% to 2.3% better. LR models produce a negative to positive difference in comparison with the one-stage model, which may suggest that LR models are not as robust as SVM and ANN models.

## 4. Conclusions

Corn syrup is a cost-effective sweetener ingredient for the food industry. Process control to enhance and/or maintain a constant dextrose equivalent value (DE) during the corn-to-syrup process is needed. Existing work has focused on continuous process control to keep parameter values within a setpoint using time series data. There has been relatively little work on building process control models to enable semi-automation in small to medium-size factories where data are not time dependent. In this paper, the corn-to-syrup process was divided into two stages. The emphasis in Stage I is on controlling the pH value and the emphasis in Stage II is on controlling the DE. Correlation coefficients were used to identify key process parameters that contribute to the feed pH value in Stage I and DE in Stage II. Artificial neural network, support vector machine, and linear regression models were built to model Stages I and II. The results suggest that (1) model accuracy yields ranged from 91% to 96%; (2) the ANN models yielded about 1% to 3% higher accuracy than the SVM and LR models, and the prediction accuracy is robust even with as few as six data sets; (3) both the SVM and ANN models have noise-tolerant properties, but ANN has a higher noise tolerance than SVM; (4) SVM performance can be hindered when using high-dimensional data sets; (5) the LR model yields a higher variation in accuracy prediction than ANN and SVM; (6) distribution fitting is a good approach for generating data; however, the fidelity of fitting can greatly impact the accuracy; and lastly, (7) multi-stage models yield higher accuracy than single-stage models, though there are pros and cons for each approach.

Technically, we expect that implementing these models in a real-time fully automated production environment should be relatively straightforward. An industrial controller would need to be embedded with an ANN model to fuse sensory information from a sensor network and make appropriate adjustments to process parameters to enhance performance measures such as the DE.

This work can potentially be used by small to medium-size companies to improve corn syrup yield and quality and to make their production processes more efficient. It is estimated that these companies produce approximately 15% of the corn syrup in the U.S., which works out to about 150,000 metric tons annually. It is also potentially applicable to other continuous process-oriented settings such as soft drink production.

Future work includes generalizing the models by testing them on a separate data set or applying them in a different production setting. We may also develop an integrated modeling approach in which regression models are developed for production processes at different levels—e.g., pilot, semi-automated, and continuous processes—as more data sets become available and data sets become more time dependent.

## Figures and Tables

**Table 1:** Correlation of each Stage I parameter with Stage I performance measure (FeedpHLab).

Stage I Parameter	Correlation with FeedpHLab
water_pH	−0.324
baume	0.212
_starch_flow	0.136
_water_flow	−0.109
_drain_flow	−0.101
conductivity	−0.077
_acid_flow	−0.075
_feed_pH_sen.	0.067
_tank_level	0.027
_starch_pH	−0.012

**Table 2:** Correlation of each Stage II parameter with Stage II performance measure (DE).

Stage II Parameter	Correlation with DE
_heat2	0.954
_heat1	0.890
_recirc_pump_amps	−0.371
_feed_ph_lab	0.156
_feed_ph_sen	−0.088
_tank_level	0.041

**Table 3:** Experiments to identify parameters for Stage I model.

Parameter	I (10)	II (9)	III (8)	IV (7)	V (6)	VI (5)	VII (4)	VIII (3)	IX (2)	X (1)	µ	SD
water_pH	X	X	X	X	X	X	X	X	X	X		
Baume	X	X	X	X	X	X	X	X	X			
_starch_flow	X	X	X	X	X	X	X	X		X		
_water_flow	X	X	X	X	X	X	X					
_drain_flow	X	X	X	X	X	X						
Conductivity	X	X	X	X	X							
_acid_flow	X	X	X	X								
feed_pH_sen	X	X	X									
_tank_level	X	X										
_starch_pH	X											
ANN Acc %	93.5	94.9	94.8	95.5	95.0	91.7	95.4	95.2	95.8	96.2	94.8	1.31
SVM Acc %	96.3	96.2	96.2	96.2	96.2	96.1	96.0	95.8	95.7	95.9	96.1	0.19
LR Acc %	93.8	93.9	96.1	96.7	96.7	96.7	96.7	96.7	96.8	96.8	96.1	1.21

**Table 4:** Experiments to identify parameters for Stage II model.

	Experiment (No. of Parameters)										
Parameter	I (6)	II (5)	III (4)	IV (3)	V (2)	VI (5)	VII (4)	VIII (3)	IX (3)	µ	SD
Heat 2	X	X	X	X	X	X	X	X	X		
Heat 1	X	X	X	X	X	X	X	X	X		
Tank Level	X	X	X	X		X					
Recirc	X	X	X								
Feed pH lab	X	X				X	X		X		
Feed pH Sen.	X					X	X	X			
ANN Acc %	94.2	94.5	93.5	93.9	93.7	94.7	94.7	94.1	93.9	94.13	0.41
SVM Acc %	92.2	92.0	93.3	92.6	93.8	93.5	93.5	94.0	93.1	93.11	0.66
LR Acc %	89.8	93.0	93.0	92.4	92.6	89.1	91.6	91.9	92.8	91.80	1.41

**Table 5:** Prediction accuracy of SI_ANN_ and SI_SVM_ models for different sample sizes.

	6	9	10	15	23	53	153	253	353	µ	SD
SI_ANN_	97.7	97.52	97.58	96.84	95.13	96.06	96.31	96.65	96.17	96.39	0.70
SI_SVM_	97.8	97.6	97.67	96.83	97.42	95.71	96.27	95.59	95.92	96.49	0.77

**Table 6:** Prediction accuracy of SII_ANN_ and SII_SVM_ models for different sample sizes.

	6	9	10	15	23	53	153	253	353	µ	SD
SII_ANN_	94.5	86.6	97.30	96.12	96.22	95.05	95.71	95.03	95.19	95.80	0.76
SII_SVM_	93.1	94.3	97.65	96.82	97.40	95.60	96.34	95.81	96.02	96.52	0.73

**Table 9:** Effect of parameters on performance measure (DE).

Para.	15	14	13	12	11	10	9	µ	SD
ANN	93.78	94.09	93.82	93.52	93.14	93.98	94.42	93.70	0.76
SVM	79.27	80.33	82.46	82.71	83.84	85.93	88.72	87.35	5.23
LR	93.44	92.69	93.14	93.07	94.08	94.32	92.67	93.30	0.61
Para.	8	7	6	5	4	3		Corr	
ANN	93.73	91.41	93.89	94.28	93.93	93.73		0.08	
SVM	90.73	92.21	92.92	93.40	94.15	90.73		−0.99	
LR	94.12	92.97	92.23	93.39	93.43	94.12		0.05	

**Table 10:** Comparison of accuracy for multi-stage versus one-stage models.

Para.	Stage I	Stage II	One Stage	Difference
ANN	96.2	94.7	93.9	0.8–2.3%
SVM	95.9	93.5	92.9	0.6–3.0%
LR	96.8	91.6	92.2	−0.6–4.6%

## Data Availability

Restrictions apply to the availability of these data. Data were obtained from a third party and permission is needed to release their data.
